# Crossed Andreev reflection in zigzag phosphorene nanoribbon based ferromagnet/superconductor/ferromagnet junctions

**DOI:** 10.1038/s41598-022-10086-2

**Published:** 2022-04-12

**Authors:** Ruigang Li, Lei Chen, Jun-Feng Liu, Jun Wang

**Affiliations:** 1 Department of Physics, School of Physics and Materials Science, Guangzhou University, Guangzhou, 510006 China; 2 College of Science, Beijing University of Civil Engineering and Architecture, Beijing, 100044 China; 3 Department of Physics, Southeast University, Nanjing, 210096 China

**Keywords:** Applied physics, Condensed-matter physics, Electronics, photonics and device physics

## Abstract

We study the crossed Andreev reflection in zigzag phosphorene nanoribbon based ferromagnet/superconductor/ferromagnet junction. Only edge states, which are entirely detached from the bulk gap, involved in the transport processes. The perfect crossed Andreev reflection, with the maximal nonlocal conductance $-2e^{2}/h$, is addressed by setting the electric potentials of the leads and device properly. At this situation, the local Andreev reflection and the electron tunneling are completely eliminated, the incoming electrons can only be reflected as electrons or transmitted as holes, corresponding to the electron reflection and the crossed Andreev reflection respectively.The nonlocal conductance oscillates periodically with the length and the electric potential of the superconductor. Our study shows that the phosphorene based junction can be used as the quantum device to generate entangled-electrons.

## Introduction

In a superconductor junction, the nonlocal coupling between electrons from different leads produces the crossed Andreev reflection (CAR)^1^. It is desirable to find large probability and convenient manipulation of CAR in electronic devices, as the reversed process of CAR is practical in generating nonlocal entangled-electrons^2–4^. Nowadays, the normal/superconductor/normal (N/S/N) junction had been used to detect CAR^5,6^, where the superconductivity of the junction can be induced by the proximity effect. At the same time, the discovery of graphene^7–10^ become a breakthrough in the field of two-dimensional material. Studies of CAR in graphene based superconductor junctions were reported since then. Cayssol^11^ proposed a graphene based N/S/N junction and point out that the nonlocal conductance acts as a function of the length of the narrow superconductor region. Wu et al.^12^ found that a cooper pair split into two nonlocal electrons perfectly in a superconductor junction based on bilayer graphene. Besides spin, they stressed that such two electrons are also involved in valley entanglement. Beiranvand et al.^13^ provided a new way to create entangled-electrons with same spin by studying the anomalous CAR in a graphene based junction, where the device is consist of a triplet pairing superconductor. In Ref.^14^, researchers examine the CAR in EuO-graphene/superconductor/EuO-graphene junction, where the lead and device region would be induced to a ferromagnet and superconductor by the proximity effect. They found that the pure CAR oscillates rapidly and periodically with the length of the device. Meanwhile, study of CAR in graphene nanoribbon junctions was reported. Zhang et al.^15^ proposed a ferromagnetic/superconductor junction based on zigzag graphene nanoribbon, and pointed out that the CAR in such junction is controllable by changing the direction of the ensemble magnet.

Recently, phosphorene^16–21^, a graphene-like two-dimensional material, had been successfully isolated. Like graphene, the honeycomb structure of phosphorene nanoribbon provides two distinct nanoribbons: armchair and zigzag nanoribbon. The armchair nanoribbon is a gapped semiconductor, while the zigzag one contains four degenerate quasi-flat edge bands detached from the bulk bands, considering spin degeneracy^21^. Generally, the electronic properties of zigzag phosphorene nanoribbon (ZPR) are dominated by the edge bands. Because the electrons in the edge bands are approached to the edges, it can be easily controlled by an external field, especially by the electric field^19^. Furthermore, the edge bands are entirely detached from the bulk bands, which helps them to eliminate the trouble caused by the bulk bands. Such magnificent properties of edge bands make it good for building the electronic device. Note that the Josephson effect in phosphorene based superconductor junctions were investigated^20^. Researcher found the supercurrent is highly anisotropic, due to the anisotropic band structure of the system. Furthermore, the supercurrent is also affected by the electric potential and the length of the superconductor region.

However, compared to graphene, according to our investigation, the study of CAR in phosphorene based junction is still a few.In this Letter, we study the crossed Andreev reflection in zigzag phosphorene nanoribbon based ferromagnet/superconductor/-ferromagnet (F/S/F) junction. The ferromagnetism and the superconductivity of phosphorene nanoribbon can be achieved in proximity to EuO^22^ and s-wave superconductor respectively. According to the first principle calculation, the exchange energy can reach 184 meV. Normally, the electron tunneling (ET) and local Andreev reflection (LAR) will compete with the CAR in the scattering processes inevitably^23^, making the nonlocal conductance weaken. In our study, the scattering processes in the junction can be consisted of CAR and electron reflection (ER), where the ET and LAR are completely blocked by setting the electric potentials of both leads and device properly. The maximal nonlocal conductance of CAR can reach $-2e^{2}/h$, corresponding to a perfect CAR. Also, we found out that the nonlocal conductance is oscillating periodically with the length and electric potential of the superconductor. Our finding shows that the phosphorene based junction can be used as the quantum device to generate entangled-electrons.

This work is organized as follows. The structure and model of the junction are presented in “Structure and model” section; the calculation results and the corresponding analysis are presented in “Results and discussion” section. In “Conclusion” section, the conclusion of this work is given.

## Structure and model

**Figure 1:**
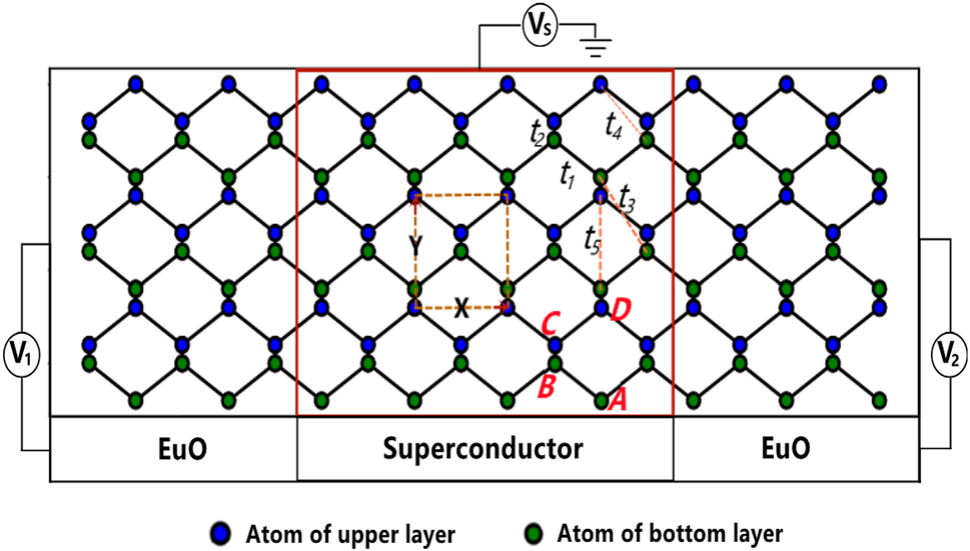
Schematic of the ferromagnet/superconductor/ferromagnet junction based on zigzag phosphorene nanoribbon.

Figure 1 is the structure we studied. The F/S/F junction is based on ZPR, with the superconductor connected to two ferromagnetic leads. The ferromagnet and superconductor can be achieved by the proximity effect. The primitive cell is shown in the regular frame with the size $X \times Y$, where *X* = 3.27 Å and *Y* = 4.43 Å^18^ are the length of the cell in x and y direction respectively. The size of the device region is $L \times T$ = $N_{L}X \times N_{T}Y$, where $N_{L}$ and $N_{T}$ denote the number of unit cell in x and y direction respectively. For a convenient description, $N_{L}$ and $N_{T}$ are referred to as the length and the width of the device region respectively, the width of leads is the same as device region. Four atoms in a unit cell are labelled as A,B,C and D. A and B(C and D) belong to the bottom(upper) layer of the phosphorene nanoribbon. As we know, the effective Hamiltonian of the bare phosphorene nanoribbon can be written in a tight binding form

$$\begin{aligned} H_{0\sigma } = \sum _{i}t_{1}(c_{A_{i}\sigma }^{\dagger }c_{B_{i}\sigma } +c_{C_{i}\sigma }^{\dagger }c_{D_{i}\sigma })+t_{2}(c_{A_{i} \sigma }^{\dagger }c_{D_{i}\sigma }+c_{B_{i}\sigma }^{\dagger }c_{C_{i}\sigma }) +\sum _{\left\langle \ i,j \right\rangle \ }t_{4}(c_{i\sigma }^{\dagger }c_{j\sigma })+H.c. \end{aligned}$$

where $c_{A_{i}\sigma }$ ( $c_{B_{i}\sigma }$, $c_{C_{i}\sigma }$, $c_{D_{i}\sigma }$) denotes annihilating an electron on site $A_{i}$ ($B_{i}, C_{i}, D_{i}$) with spin $\sigma = \uparrow /\downarrow$. There are 5 hopping parameters in $H_{0\sigma }$, shown in Fig. 1. The parameters read as $t_{1} = -1.220\,\hbox {eV}$, $t_{2} = 3.665 \,\hbox {eV}$, $t_{3} =-0.205\,\hbox {eV}$, $t_{4} = -0.105\,\hbox {eV}$, and $t_{5} = 0.055 \,\hbox {eV}$^24^. Actually, compare with the model in Ref.^24^, $t_{3}$ and $t_{5}$ are neglected, because they bring no important effect on the transport properties of phosphorene nanoribbon. $\langle i, j\rangle$ in $H_{0\sigma }$ denotes that an electron or a hole hops from atom *j* in one layer to atom *i* in another layer,shown in Fig. 1.

Bogliubov-de Gennas (BdG) equation is used to study the CAR in ZPR superconductor junction. The tight-binding Hamiltonian of the junction can be written as

$$\begin{aligned} & H = H_{S}[\Theta (x)-\Theta (x-L)]+H_{F1}\Theta (-x)+H_{F2}\Theta (x-L)+H_{T} \\&H_{1\uparrow / \downarrow }=H_{0 \uparrow / \downarrow } \pm \varepsilon _{\mathrm {ex}} \\&H_{T}=\sum _{< F, S >} {\mathrm {t}}_{1} {\mathrm {c}}_{\mathrm {S}}{ }^{+} {\mathrm {c}}_{\mathrm {F}}+H . c \\&H_{S}=\left( \begin{array}{*{20}l} H_{0 \uparrow }+V_{s} &{}\quad 0 &{}\quad 0 &{}\quad \Delta \\ 0 &{}\quad H_{0 \downarrow }+V_{s} &{}\quad -\Delta &{}\quad 0 \\ 0 &{}\quad -\Delta ^{*} &{}\quad -V_{s}-H_{0 \uparrow } &{}\quad 0 \\ \Delta ^{*} &{}\quad 0 &{}\quad 0 &{}\quad -V_{s}-H_{0 \downarrow } \end{array}\right) \\&H_{F1(F2)}=\left( \begin{array}{*{20}l} H_{1\uparrow }+V_{1(2)} &{}\quad 0 &{}\quad 0 &{}\quad 0 \\ 0 &{}\quad H_{1\downarrow }+V_{1(2)} &{}\quad 0 &{}\quad 0 \\ 0 &{}\quad 0 &{}\quad -V_{1(2)}-H_{1\uparrow } &{}\quad 0 \\ 0 &{}\quad 0 &{}\quad 0 &{}\quad -V_{1(2)}-H_{1\downarrow } \end{array}\right) \end{aligned}$$

$H_{T}$ stands for the couple between superconductor region and ferromagnetic leads, where $c_{S}^{\dagger }c_{F}$ represents a particle hops from the lead to the device region, and $\langle F, S\rangle$ denotes the hopping happens between nearest atoms. $H_{S}/H_{F1(F2)}$ denotes the Hamiltonian of superconductor/left(right) ferromagnet, $\Theta$ is the Heaviside step function. $\Delta$ in $H_{S}$ is the order parameter of the superconductor. $\varepsilon _{ex}$ in $H_{F1(F2)}$ represents the exchange energy in the ferromagnetic leads, and the value of $\varepsilon _{ex}$ is set to be 0.2 eV in this study. $V_{1(2)} /V_{s}$ is the electric potential of the left(right) lead/superconductor region, which can be tuned by a top gate voltage.

The calculation in this study is done by Kwant^25^, a software for quantum transport calculation. The tight-binding model of the system can be built by Kwant’s package, and the structure can be visualized by setting system parameters. In detail, the lattice built by Kwant is the phosphorene lattice shown in Fig. 1. Note that the lattice of monolayer phosphorene described by $C_{2h}$ point group. The leads belong to the same lattice and they are semi-infinite. After that, the scattering matrix [*S*] can be obtained. In our study, the expression of the wave function in the right lead is $\sum _{e} S_{e h}\left( E_{F}\right) \psi _{e}$, where e denotes the injecting electrons in the left lead, h denotes the outgoing holes in the right lead and $E_{F}$ is the Fermi energy. $S_{eh}$ denotes the element of scattering matrix, and the transmission can be written as $T_{e h}=\left| S_{e h}\left( E_{F}\right) \right| ^{2}$. For simplicity, we can simply just consider the CAR process form the left lead to the right lead^11,14,26,27^. According to the Ref.^26^, the nonlocal conductance can be given as

$$\begin{aligned} {\mathrm {G}}=\left[ \frac{d I_{R}}{d V_{L}}\right] _{V_{R}=0}=-\frac{\mathrm {e}^{2}}{\mathrm {~h}} {\mathrm {~T}}_{e h} \end{aligned}$$

where $I_{R}$ can be measured as the current formed in the right lead and $V_{L}$ is the bias applied between the left lead and the device. Kwant uses the efficient method of solving linear equations, making the calculation speed faster than the lattice Green function approach. The wavefunctions and the band structures of the leads can be obtained. For more details please refer to Ref.^25^.

## Results and discussion

**Figure 2:**
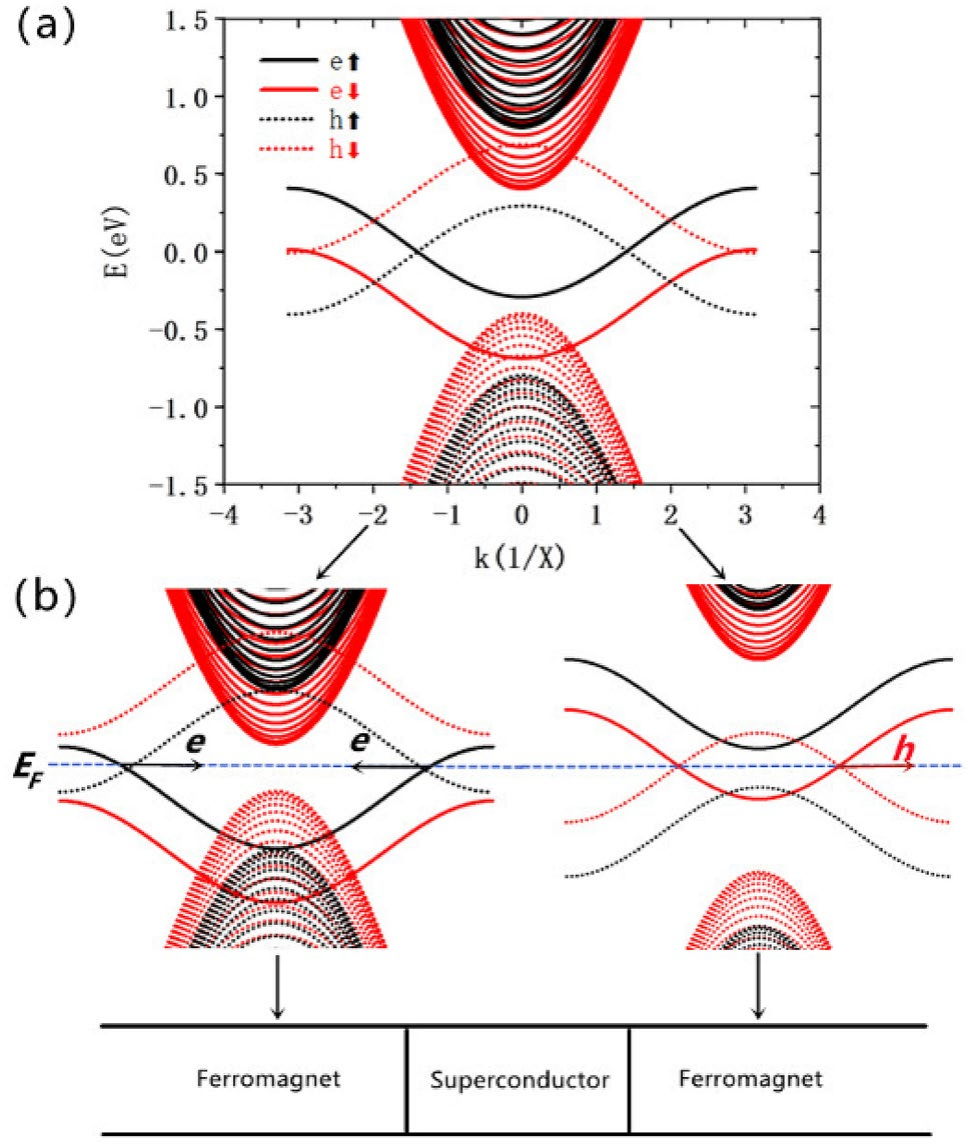
(**a**) The band structure of the ferromagnetic lead with $V = 0 \,\hbox {eV}$, $N_{T} = 50$. (**b**) Band structures of left and right ferromagnetic lead with $V_{1}< 0$ and $V_{2}> 0$ respectively. The band structures are computed by the KWANT package.

The band structures of the ferromagnetic lead had been shown in Fig. 2. The electron and hole bands are drawn together, which are plot in solid and dot lines respectively. Note that, there exist four electron (hole) edge bands in the band gap, considering the spin degeneracy. The electron edge bands are separated into two double degenerate bands by the exchange field, which can be found in Fig. 2a. As $\varepsilon _{ex}$ is set as 0.2 eV, the electrons in upper/bottom electron edge bands are spin-up/spin-down electrons. Here, we set $V_{1}<0$ and $V_{2}>0$ to get the band structure of left and right lead in Fig. 2b respectively. According to the Hamiltonian of the ferromagnetic lead, when the extra electric potential is set to be $V<0$, the electron bands will be pushed down and the hole bands will be uplifted. By doing so, the LAR and ET can be eliminated in the scattering processes. This can be understood from Fig. 2b, at the Fermi energy $E_{F}$, there are no conducting modes to reflect holes and transmit electrons; Furthermore, suppose there is no spin flipping in the processes, the bands of spin-up hole and the spin-down electron will not contribute to the scattering processes when the spin-up electron is injecting into the system from left lead. Therefore, only two situations happen when electrons injecting into the system: reflect electrons or transmit holes for forming ER or CAR respectively.

**Figure 3:**
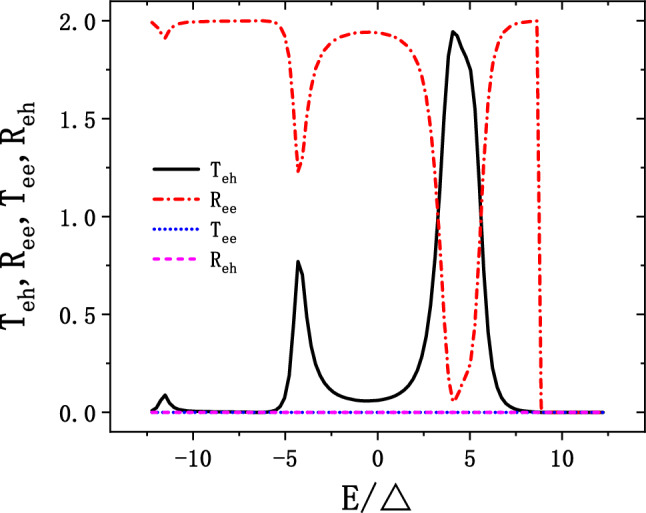
The scattering coefficients of CAR($T_{eh}$), ER($R_{ee}$), ET($T_{ee}$) and LAR($R_{eh}$), the parameters of the junction are setting as: $N_{T} = 40$, $N_{L} = 40$, $V_{s} = -0.164 \,\hbox {meV}$, $V_{1} = -0.4 \,\hbox {eV}$, $V_{2} = 0.6 \,\hbox {eV}$.

The calculation results are shown in Fig. 3. The number of unit cell of the device region are $N_{L} = 40$, $N_{T} = 40$. Without losing generality, the electric potential of left (right) lead is set to be $V_{1} = -0.4 \,\hbox {eV}$ ($V_{2} = 0.6 \,\hbox {eV}$), so that there is only spin-up electron injecting into the system around $E = 0 \,\hbox {eV}$. The electric potential and the superconductor gap of the superconductor are set to be $V_{s} = -164 \,\hbox {meV}$ and $\Delta = 1.5 \,\hbox {meV}$ respectively. Note that, the scattering coefficients $T_{eh}$, $R_{ee}$, $T_{ee}$ and $R_{eh}$ denote CAR, ER, ET and LAR process respectively. Agree with our analysis above, the scattering coefficients of LAR and ET are zero, i.e., $R_{eh} = 0$ and $T_{ee} = 0$, for there is no conducting modes for these two processes around $E = 0 \,\hbox {eV}$. Since there are two electrons injecting into the junction simultaneously and only CAR and ER existed in the scattering processes at the energy range we studied, the relation $T_{eh} + R_{ee} = 2$ is obtained. The scattering coefficients $T_{eh}$ and $R_{ee}$ in Fig. 3 are asymmetric with respect to the E coordinate. Two peaks of $T_{eh}$ appear at $E = -4\Delta$ and $E = 4\Delta$ in Fig. 3, with the values 0.75 and 2 respectively. Note that, the maximal $T_{eh}$ can achieve 2 at $E = 4\Delta$, corresponding to the nonlocal conductance $-2e^{2}/h$. Such phenomenon is referred to as the perfect CAR, where the incoming electrons from one lead are entirely converting to outgoing holes in another lead.

**Figure 4:**
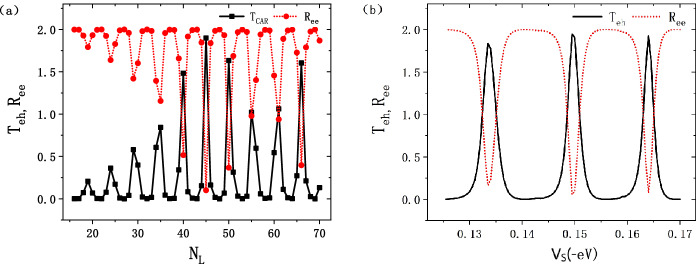
The scattering coefficients of CAR and ER as a function of (**a**) the length ($N_{L}$) and (**b**) the electric potential ($V_{s}$) of the superconductor, with $\hbox {E} = 2.16\Delta$.

Figure 4 shows the scattering coefficients of CAR (and ER) as a function of the length $N_{L}$ and the electric potential $V_{s}$ of superconductor. By the way, as the change of $N_{T}$ of the junction will barely affect the band gap and $T_{eh}$, we defaultly fixed $N_{T} = 40$ in this study. The calculation results are shown in Fig. 4. In Fig. 4a, $T_{eh}$ as a function of $N_{L}$, with $V_{s}$ and *E* are fixed at $-149 \,\hbox {meV}$ and $2.16\Delta$ respectively. As we see in Fig. 4a, the $T_{eh}$ and $R_{ee}$ are oscillating periodically with $N_{L}$, with the period $\Delta N_{L} = 5$. The peak’s value of $T_{eh}$ is changing with $N_{L}$. We also found that there exists a peak at $N_{L}= 45$, with $T_{eh} = 2$, in Fig. 4a.

Then, when we change $V_{s}$ and fix $N_{L} = 40$ and $E = 2.16\Delta$, a similar effect happens. The $T_{eh}$ oscillates with a period $\Delta V_{s} = 15 \,\hbox {meV}$, which means that the CAR is quite sensitive to the change of electric potential of the superconductor. We also found $T_{eh} = 2$ around $V_{s} = -164 \,\hbox {meV}$. The perfect CAR in Fig. 4 is attributed to the resonance effect of the quantum transport, the resonance condition is $kL=n\pi$, *k* and *L* are the wave vector and the length of the device region respectively. The mechanism of such periodic osocillation had reported in Ref.^28^. These properties make the CAR in the junction controllable.

**Figure 5:**
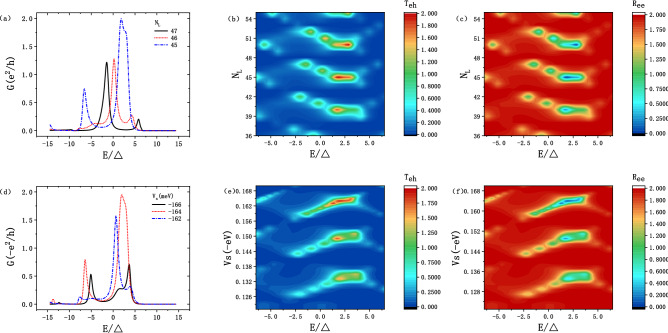
(**a**) The nonlocal conductance for $N_{L}$ = 45, 46 and 47. (**b**)/(**c**) The transmission/reflection of CAR/ER as a function of $N_{L}$ and the energy *E*, with $V_{s} =-149 \,\hbox {meV}$. (**d**) The nonlocal conductance for $V_{s} = -162$, $-164$ and $-166 \,\hbox {meV}$. (**e**)/(**f**) The transmission/reflection of CAR/ER as a function of $V_{s}$ and the energy *E*, with $N_{L}=40$.

The nonlocal conductance are presented at Fig. 5a and d. In Fig. 5a, when $N_{L}$ increases from 45 to 47, the CAR peak moves from the higher energy to a lower one, with the $N_{L}$ changing. Such phenomenon also happens in changing $V_{s}$, which is shown in Fig. 5d. These properties can be easily found in Fig. 5b and e. When the energy is in the range $[-7.5\Delta , 7.5\Delta ]$, there is a conductance “gap” at $N_{L} = 43/48/53$ and $V_{s}$ around $-138/-153/-168 \,\hbox {meV}$, the corresponding $T_{eh}$ are suppressed and close to zero. Considering Fig. 5b/e and c/f, the $T_{eh}$ and $R_{ee}$ are complementary, with $T_{eh} + R_{ee} = 2$.

## Conclusion

In this work, we studied the crossed Andreev reflection in the ferromagnet/superconductor/ferromagnet junction based on zigzag phosphorene nanoribbon. By setting the electric potential of both leads differently, the scattering processes consists of electron reflection and crossed Andreev reflection, where the local Andreev reflection and electron tunneling are suppressed. The maximal nonlocal conductance can reach $-2e^{2}/h$, which is referred to as the perfect CAR. We also found that the transmission coefficient of crossed Andreev reflection oscillates periodically with the length and the electric potential of the superconductor. These properties make the CAR controllable. Our finding will be helpful to devise the quantum device for generating entangled-electrons.

## References

[1] Byers JM, Flatté ME (1995). Probing spatial correlations with nanoscale two-contact tunneling. Phys. Rev. Lett..

[2] Samuelsson P, Sukhorukov EV, Büttiker M (2005). Quasi-particle entanglement: Redefinition of the vacuum and reduced density matrix approach. New J. Phys..

[3] Prada E, Sols F (2004). Entangled electron current through finite size normal-superconductor tunneling structures. Eur. Phys. J. B.

[4] Lesovik GB, Martin T, Blatter G (2001). Electronic entanglement in the vicinity of a superconductor. Eur. Phys. J. B.

[5] Islam SK, Dutta P, Saha A (2017). Enhancement of crossed Andreev reflection in a normal-superconductor-normal junction made of thin topological insulator. Phys. Rev. B.

[6] Celis Gil JA, Gomez PS, Herrera WJ (2017). Noise cross-correlation and Cooper pair splitting efficiency in multi-teminal superconductor junctions. Solid State Commun..

[7] Castro Neto AH, Guinea F, Peres NM, Novoselov KS, Geim AK (2009). The electronic properties of graphene. Rev. Mod. Phys..

[8] Geim AK, Novoselov KS (2007). The rise of graphene. Nat. Mater..

[9] Ferrari AC (2006). Raman spectrum of graphene and graphene layers. Phys. Rev. Lett..

[10] Lee C, Wei X, Kysar JW, Hone J (2008). Measurement of the elastic properties and intrinsic strength of monolayer graphene. Science.

[11] Cayssol J (2008). Crossed Andreev reflection in a graphene bipolar transistor. Phys. Rev. Lett..

[12] Wu X (2020). Tunable nonlocal valley-entangled Cooper pair splitter realized in bilayer-graphene van der Waals spin valves. Phys. Rev. B.

[13] Beiranvand R, Hamzehpour H, Alidoust M (2017). Nonlocal Andreev entanglements and triplet correlations in graphene with spin-orbit coupling. Phys. Rev. B.

[14] Ang YS, Ang LK, Zhang C, Ma Z (2016). Nonlocal transistor based on pure crossed Andreev reflection in a EuO-graphene/superconductor hybrid structure. Phys. Rev. B.

[15] Zhang L, Tian HY, Wang J (2012). Nonlocal conductance control of a zigzag F/S/F graphene nanoribbon junction. EPL.

[16] Li L (2014). Black phosphorus field-effect transistors. Nat. Nanotechnol..

[17] Jia Y, Xia F, Wang H (2014). Layered material for optoelectronics and electronics. Nat. Commun..

[18] Castellanos-Gomez A (2014). Isolation and characterization of few-layer black phosphorus. 2D Mater..

[19] Zhou B, Zhou B, Zhou X, Zhou G (2017). Even-odd effect on the edge states for zigzag phosphorene nanoribbons under a perpendicular electric field. J. Phys. D Appl. Phys..

[20] Linder J, Yokoyama T (2017). Anisotropic Andreev reflection and Josephson effect in ballistic phosphorene. Phys. Rev. B.

[21] Peng X (2014). Phosphorene nanoribbons. Europhys. Lett..

[22] Chen H, Li B, Yang J (2017). Proximity effect induced spin injection in phosphorene on magnetic insulator. ACS Appl. Mater. Interfaces.

[23] Falci G, Feinberg D, Hekking FW (2001). Correlated tunneling into a superconductor in a multiprobe hybrid structure. Europhys. Lett..

[24] Rudenko AN, Katsnelson MI (2014). Quasiparticle band structure and tight-binding model for single- and bilayer black phosphorus. Phys. Rev. B Condens. Matter Mater. Phys..

[25] Groth, C. W., Wimmer, M., Akhmerov, A. R. & Waintal, X. Kwant : A software package for quantum transport scattering region lead 0 lead 2 lead 1. 1–19 (2014).

[26] Zhang SB, Trauzettel B (2019). Perfect crossed Andreev reflection in dirac hybrid junctions in the quantum hall regime. Phys. Rev. Lett..

[27] Dong ZC, Dong ZC, Shen R, Zheng ZM, Xing DY, Wang ZD, Wang ZD (2003). Coherent quantum transport in ferromagnet/superconductor/ferromagnet structures. Phys. Rev. B Condens. Matter Mater. Phys..

[28] Božović M, Radović Z (2002). Coherent effects in double-barrier ferromagnet/superconductor/ferromagnet junctions. Phys. Rev. B Condens. Matter Mater. Phys..

